# A Comprehensive Prognostic and Immune Analysis of Ferroptosis-Related Genes Identifies SLC7A11 as a Novel Prognostic Biomarker in Lung Adenocarcinoma

**DOI:** 10.1155/2022/1951620

**Published:** 2022-04-25

**Authors:** Li Qian, Fu Wang, Shu-min Lu, Hua-jie Miao, Xin He, Jia Feng, Hua Huang, Rong-feng Shi, Jian-guo Zhang

**Affiliations:** ^1^Department of Pathology, Affiliated Hospital of Nantong University, Nantong, Jiangsu, China; ^2^Department of Traditional Chinese Medicine, Nantong Maternity and Child Health Care Hospital Affiliated to Nantong University, Nantong, Jiangsu, China; ^3^Department of Oncology, Xinhua Hospital Affiliated to Shanghai Jiao Tong University School of Medicine, Shanghai, China; ^4^Department of Interventional Radiology, Affiliated Hospital of Nantong University, Nantong, Jiangsu, China

## Abstract

Lung adenocarcinoma (LUAD) is still one of the illnesses with the greatest mortality and morbidity. As a recently identified mode of cellular death, the activation of ferroptosis may promote the effectiveness of antitumor therapies in several types of tumors. However, the expression and clinical significance of Ferroptosis-associated genes in LUAD are still elusive. The RNA sequencing data of LUAD and relevant clinical data were downloaded from The Cancer Genome Atlas (TCGA) datasets. Subsequently, potential prognostic biomarkers were determined by the use of biological information technology. The R software package “ggalluvial” was applied to structure Sanguini diagram. Herein, our team screened 14 dysregulated ferroptosis-associated genes in LUAD. Among them, only four genes were associated with clinical outcome of LUAD patients, including ATP5MC3, FANCD2, GLS2, and SLC7A11. In addition, we found that high SLC7A11 expression predicted an advanced clinical progression in LUAD patients. Additionally, 8 immune checkpoint genes and 7 immune cells for LUAD were recognized to be related to the expression of SLC7A11. KEGG assays indicated that high expression of SLC7A11 might participate in the modulation of intestinal immune network for IgA generation and Staphylococcus aureus infection. Overall, our findings revealed that SLC7A11 might become a potentially diagnostic biomarker and SLC7A11 might serve as an independent prognosis indicator for LUAD.

## 1. Introduction

Pulmonary carcinoma is the leading cause of tumor-related death across the globe [1]. Lung adenocarcinoma (LUAD) predominantly arises from small airway epitheliums and type II alveolar cells, accounting for 45% of lung cancer [2, 3]. The progresses in the therapy of NSCLC sufferers, like surgery, radiotherapy, chemical therapy, targeted treatment, and immune checkpoint suppressor treatment, have been reported; nevertheless, the 5-year survival is still below 15%, mainly due to cancer metastasis [4, 5]. In addition, up to now, the causal links as to the occurrence and progression of pancreas carcinoma remain elusive [6]. It is imperative to reveal potential molecule-level causal links and identify valid treatment targets and new prognosis markers for LUAD.

Recently, certain researches have explored cancer ferroptosis [7]. Ferroptosis, an iron-dependent pathway of cellular death, is different from novel types of apoptotic cellular death like apoptosis, pyrolysis, and autophagy [8, 9]. Ferroptosis is induced via the cumulation of lipoperoxidation products and cytotoxic ROS originated from Fe metabolic activity [10, 11]. Fe metabolic activity and lipoperoxidation signal transmission are identified as the predominant mediating factors of ferroptosis [12, 13]. Ferroptosis induces mitochondria function disorder and cytotoxic lipoperoxidation, which are pivotal for the inhibition of tumor progression [14, 15]. Several ferroptosis-related genes have been functionally identified [16, 17]. For instance, NRF2 modulates the susceptibility of mankind pulmonary carcinoma cells to cystine deprivation-triggered ferroptosis through the FOCAD-FAK signal path [18]. Therefore, investigating the ferroptosis-related gene expression profile and its prognostic value in LUAD may develop new strategies for the treatment of LUAD.

Herein, our team analyzed the expressing pattern of ferroptosis-associated genes using TCGA datasets and identified 24 dysregulated genes in LUAD. Then, we further explore their prognostic values. By now, there still have been few reports concerning the expression and function of SLC7A11 in human LUAD. Our attention focused on SLC7A11, and we aimed to explore the possible association between SLC7A11 expression and immune activity.

## 2. Materials and Methods

### 2.1. Data Collection

Level 3 raw counts of the RNA sequencing data, cancer mutation burden, aneuploidy scores, and relevant clinic information from an overall 513 LUAD sufferers were obtained from TCGA. Clinical variables, like sex, age, and pathology phase, were assessed as well. Ferroptosis-related genes are derived from GSEA (http://www.gsea-msigdb.org).

### 2.2. Screening of the Abnormally Expressed Ferroptosis-Related Genes in LUAD

For the sake of identifying the aberrantly expressed ferroptosis-related genes, R program 4.0.5 was leveraged to contrast the expression profiles of LUAD samples to healthy samples. DEG analyses of individual genes were completed via edgeR Bioconductor package [14]. The ∣log2 (fold change [FC]) | >2, *p* value < 0.01, and FDR < 0.01 were the liminal values for the determination of the aberrantly expressed ferroptosis-related genes.

### 2.3. Screening of Ferroptosis-Associated and Survival-Associated Genes

The association between the expressions of the aberrantly expressed ferroptosis-associated genes and survival time of LUAD was investigated. The survival function was plotted with the Kaplan-Meier plots. The entire analysis approaches aforesaid were completed via the “survival” and “survminer” R package [19]. *p* < 0.05 had significance on statistics.

### 2.4. The Association between SCL7A11 and Clinicopathological Parameters

The association between the expression of SCL7A11 and clinicopathological variables was analyzed through box plots and Sankey diagrams via independent specimen nonparametric assays in the TCGA cohort. Sankey diagrams were drawn via “ggforce” package in R.

### 2.5. TIMER Database Analysis

TIMER is a database for the analysis of immunocyte infiltrates in several tumors. Such database uses pathology test-verified statistic approach to speculate cancer immunity infiltration via neutrophilic cells, macrophages, dendritic cells, B cells, and CD4/CD8 T cells. Our team first utilized such database to evaluate diversities in SCL7A11 expressing levels in specific cancer types via the TIMER database, and our team investigated the relationship between the expression of SCL7A11 and infiltrative level via specific immunocyte subsets.

### 2.6. Gene Set Enrichment Analysis (GSEA)

GSEA was completed to analyze the biology pathway in LUAD layered via the midvalue of SCL7A11 expression. The assay was finished as per the protocol recommendation from the Broad Institute Gene Set Enrichment Analysis website. The GSEA was completed via the GSEA 4.0.3 program. NOM (*p* < 0.05) and FDR *q* < 005 (*p* < 0.05) had significance on statistics.

### 2.7. Statistical Analysis

The entire analysis was completed via R 4.0.5 and the proper packages. A two-sided *t*-test was employed to examine the association between the expression of SCL7A11 and clinic characteristics. Survival curves were drawn via the Kaplan-Meier approach and evaluated via log-rank tests. ROC curve analyses were completed for risk scoring computed on the foundation of screened genetic hallmark via survivalROC R package. Statistic assays were bilateral, with *p* ≤ 0.05 having significance on statistics.

## 3. Results

### 3.1. Twenty-Three Aberrantly Expressed Ferroptosis-Related Genes in LUAD Were Identified

**Figure 1:**
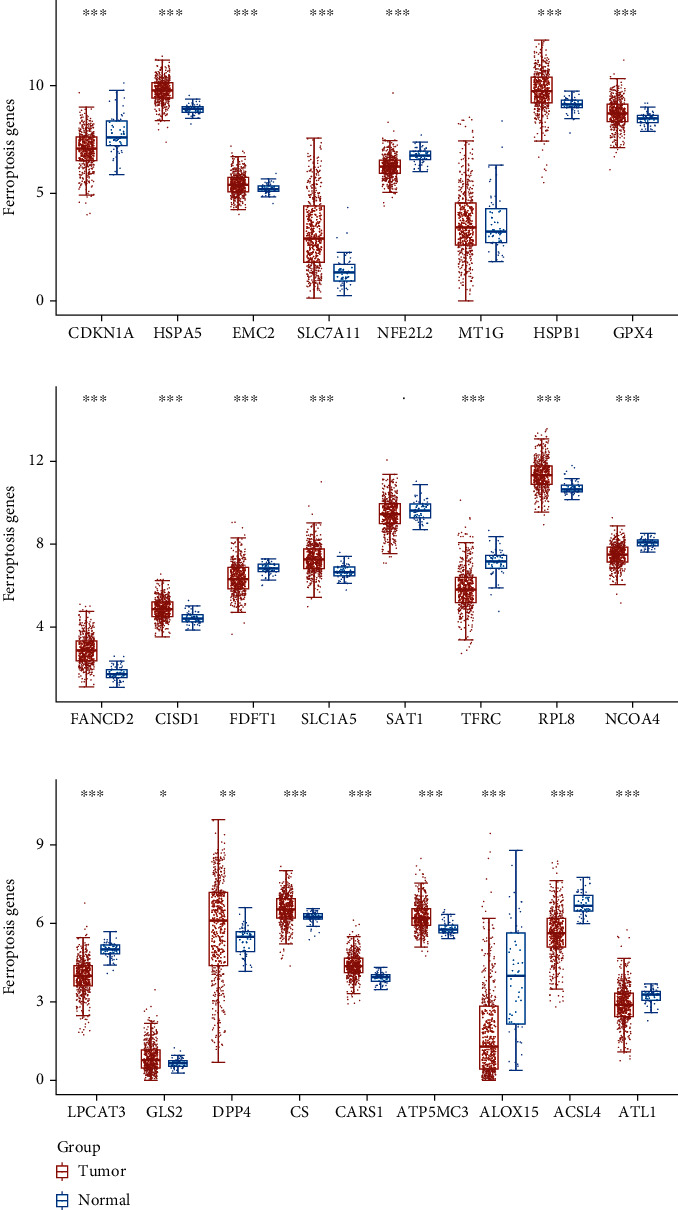
The expressing pattern of ferroptosis-associated genes in LUAD using TCGA datasets. ^∗∗∗^*p* < 0.001,  ^∗∗^*p* < 0.01, and^∗^*p* < 0.05.

To explore the possible function of aberrantly expressed ferroptosis-related genes in LUAD, our team analyzed the expressing pattern of ferroptosis-related genes via TCGA datasets. We observed that 23 ferroptosis-related genes which exhibited a dysregulated level in LUAD, such as CDKN1A, HSPA5, EMC2, SLC7A11, NFE2L2, HSPB1, GPX4, FANCD2, CISD1, FDFT1, SLC1A5, TFRC, RPL8, NCOA4, LPCAT3, DPP4, CS, CARS1, ATP5MC3, ALOX15, ACSL4, GLS2, and ACSL4 (Figure 1).

### 3.2. Identification of Survival-Related and Ferroptosis-Related Genes in LUAD

**Figure 2:**
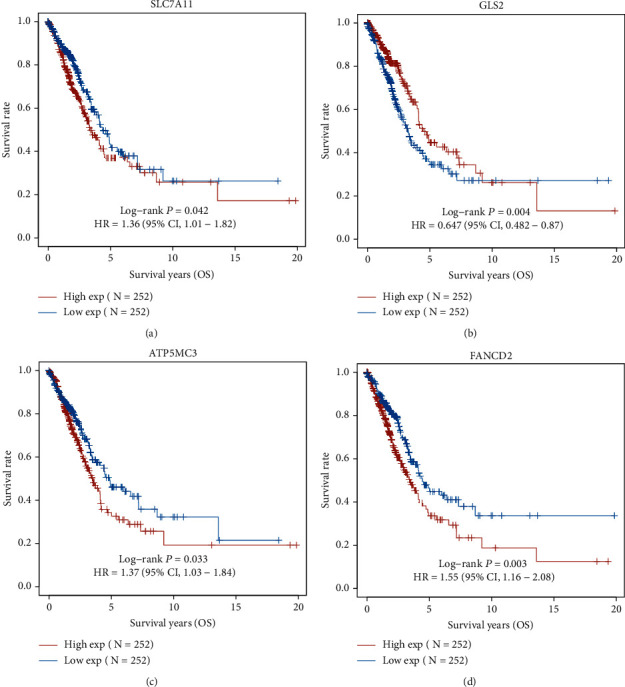
The survival-associated genes in LUAD were identified using Kaplan-Meier methods, including (a) SLC7A11, (b) GLS2, (c) ATP5MC3, and (d) FANCD2.

**Figure 3:**
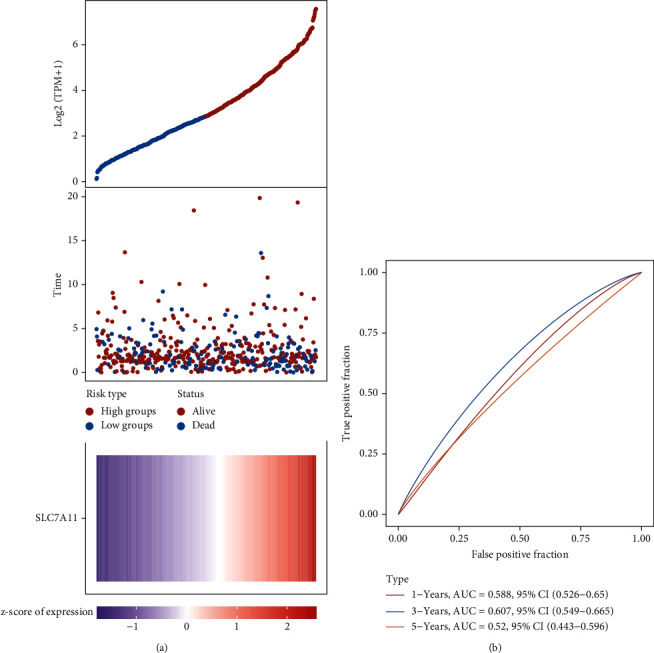
(a) The curve of risk scoring. Survival status of the patients. (b) Time-reliant ROC analyses for SLC7A11.

**Figure 4:**
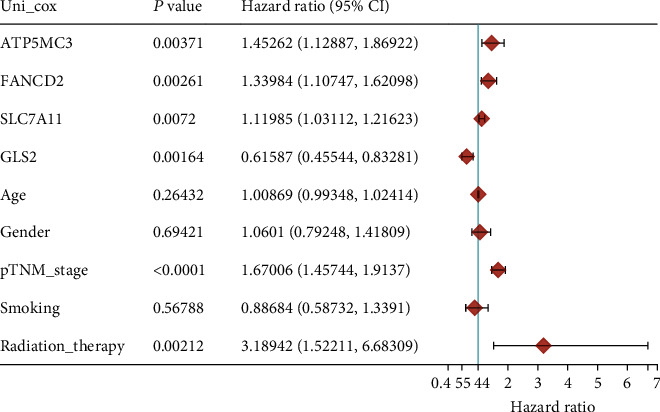
Univariable Cox regressive analyses were completed on all clinical covariates to identify its impact on survival.

**Figure 5:**
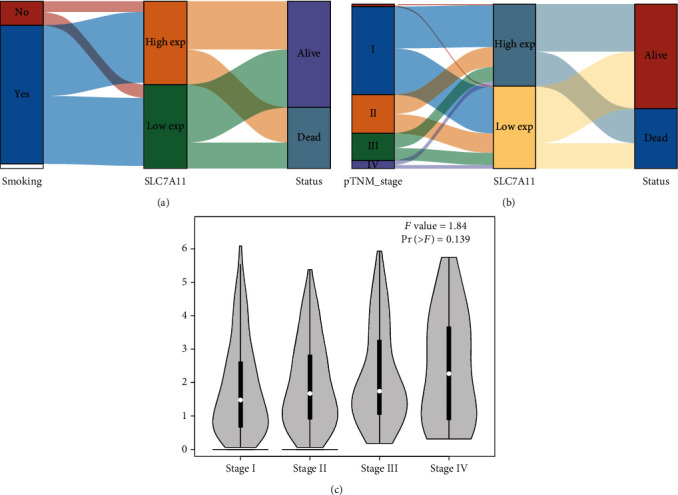
The correlation between SLC7A11 and clinicopathological parameters in LUAD. Sankey diagrams between SLC7A11 and (a) Smoking and (b) pTNM_stage. (c) High expression of SCL7A11 was identified in LUAD samples with phases III-IV in contrast to phases I-II.

Then, we performed Kaplan-Meier methods to screen survival-related and ferroptosis-related genes in LUAD. Among the above 23 aberrantly expressed ferroptosis-associated genes, we observed four genes were related to the OS of LUAD sufferers, including SLC7A11 (Figure 2(a)), GLS2 (Figure 2(b)), ATP5MC3 (Figure 2(c)), and FANCD2 (Figure 2(d)). Our attention focused on SLC7A11. The survival status of the entire LUAD sufferers is presented by Figure 3(a). Time-reliant ROC analyses revealed the prognosis accuracy was 0.588 at 1 year, 0.607 at 3 years, and 0.52 at 5 years, separately (Figure 3(b)). Moreover, univariate assays suggested the above four genes, pTNM_stage, and Radiation_therapy were risk factors for the clinical outcome of LUAD patients (Figure 4). Moreover, we performed Sankey diagrams to investigate the relationship between the expression of SCL7A11 and clinicopathological parameters, finding that high SCL7A11 predicted an advanced pTNM_stage (Figures 5(a) and 5(b)). High expression of SCL7A11 was identified in LUAD samples with phases III-IV in contrast to the phases I-II (Figure 5(c)).

### 3.3. The Relationship between the Expression of SCL7A11 and Immune Checkpoint in LUAD

**Figure 6:**
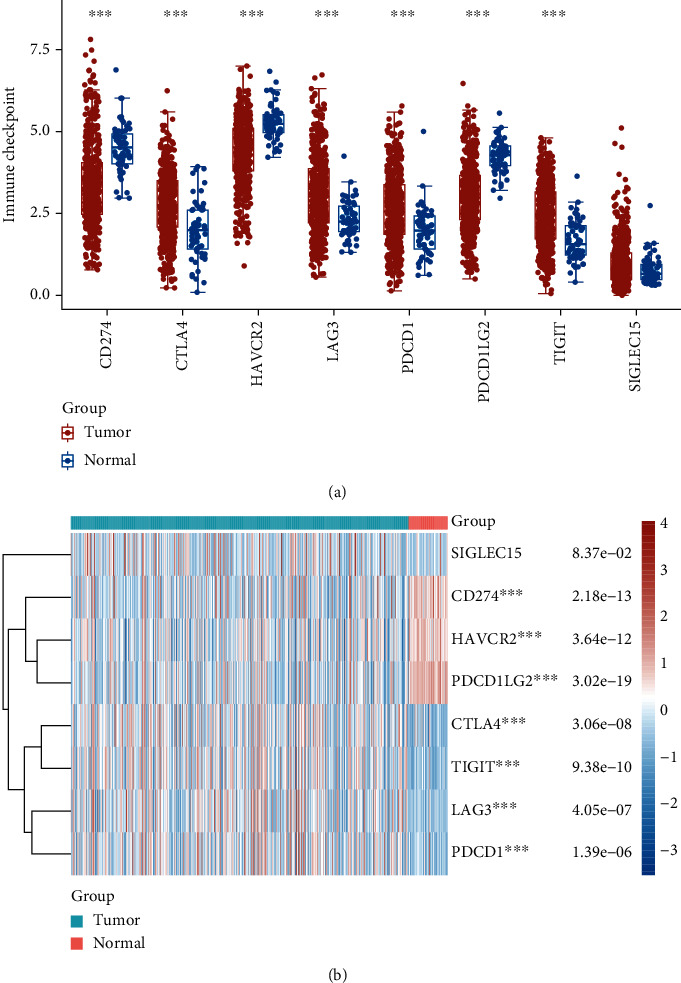
(a, b) The dysregulated expression of immune checkpoints in LUAD samples in contrast to nontumor specimens. ^∗∗∗^*p* < 0.001.

**Figure 7:**
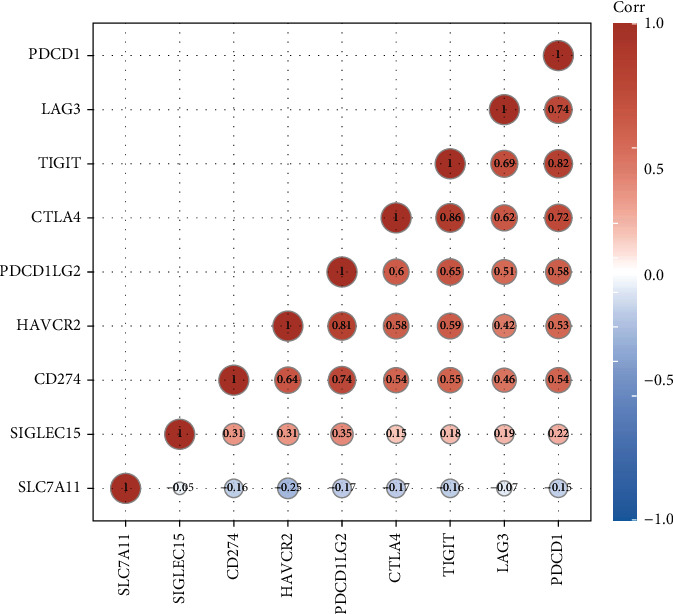
The association between SCL7A11 expression and several immune checkpoints in LUAD.

For the sake of exploring the underlying function of SCL7A11 in immunoactivity, we analyzed the expressing pattern of several immune checkpoint in LUAD. As presented by Figures 6(a) and 6(b), our team discovered that the expression of CTLA4, LAG3, PDCD1, TIGIT, and SIGLEC15 was distinctly increased in LUAD specimens in contrast to nontumor specimens, while the expression of HAVCR2, CD274, and PDCDALG2 was distinctly decreased in LUAD samples in contrast to nontumor specimens. The outcomes of correlation analyses revealed that the expression of SCL7A11 was associated with the expression of most immune checkpoints (Figure 7).

### 3.4. A Significant Correlation between SCL7A11 and Cancer-Infiltrating Immunocytes in LUAD

**Figure 8:**
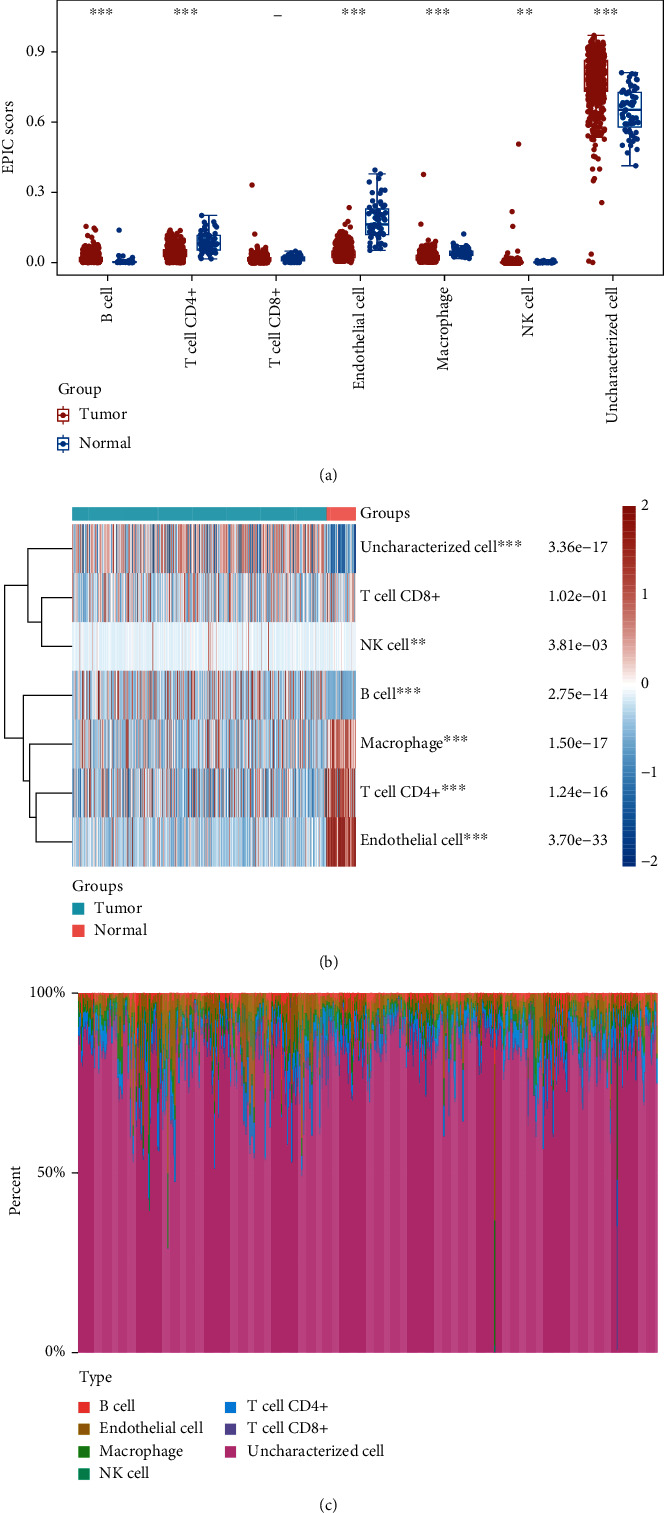
(a, b) The expressing feature of immune cells in LUAD specimens and nontumor specimens. ^∗∗∗^*p* < 0.001 and^∗∗^*p* < 0.01.

**Figure 9:**
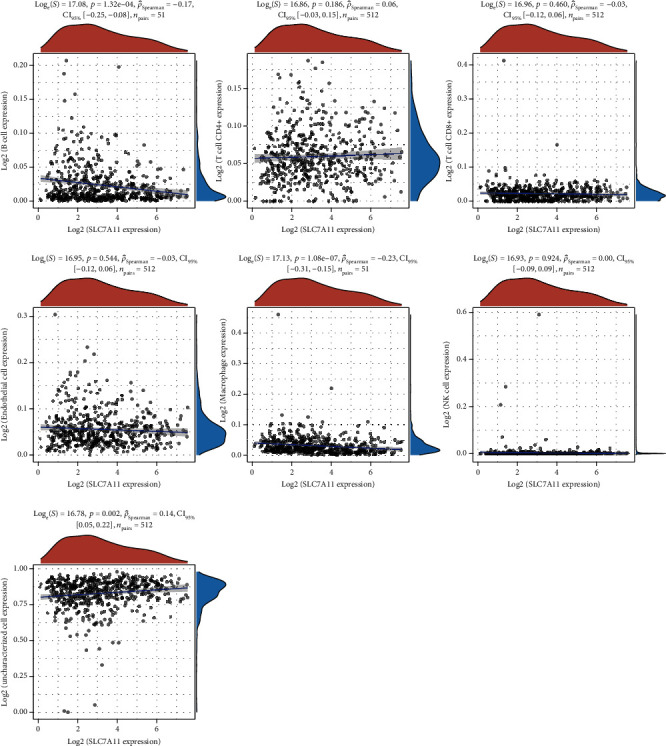
The association analysis between the expression of SCL7A11 and immunocytes.

Then, we analyzed the expression of cancer-infiltrating immunocytes in LUAD, and observed that B cell, T cell CD4+, endothelial cell, macrophages, NK cell, and uncharacterized cell exhibited a dysregulated level in LUAD samples in contrast to nontumor specimens (Figures 8(a) and 8(b)). For the sake of knowing the association between the expression of SCL7A11 and cancer-infiltrating immunocytes, the kit of TIMER was employed. A remarkable association was observed between the expression of SCL7A11 and diverse kinds of immunocytes, like B cell and macrophage (Figure 9).

### 3.5. Gene Ontology (GO) and the Kyoto Encyclopedia of Genes and Genomes (KEGG) Enrichment Analysis of DEGs

**Figure 10:**
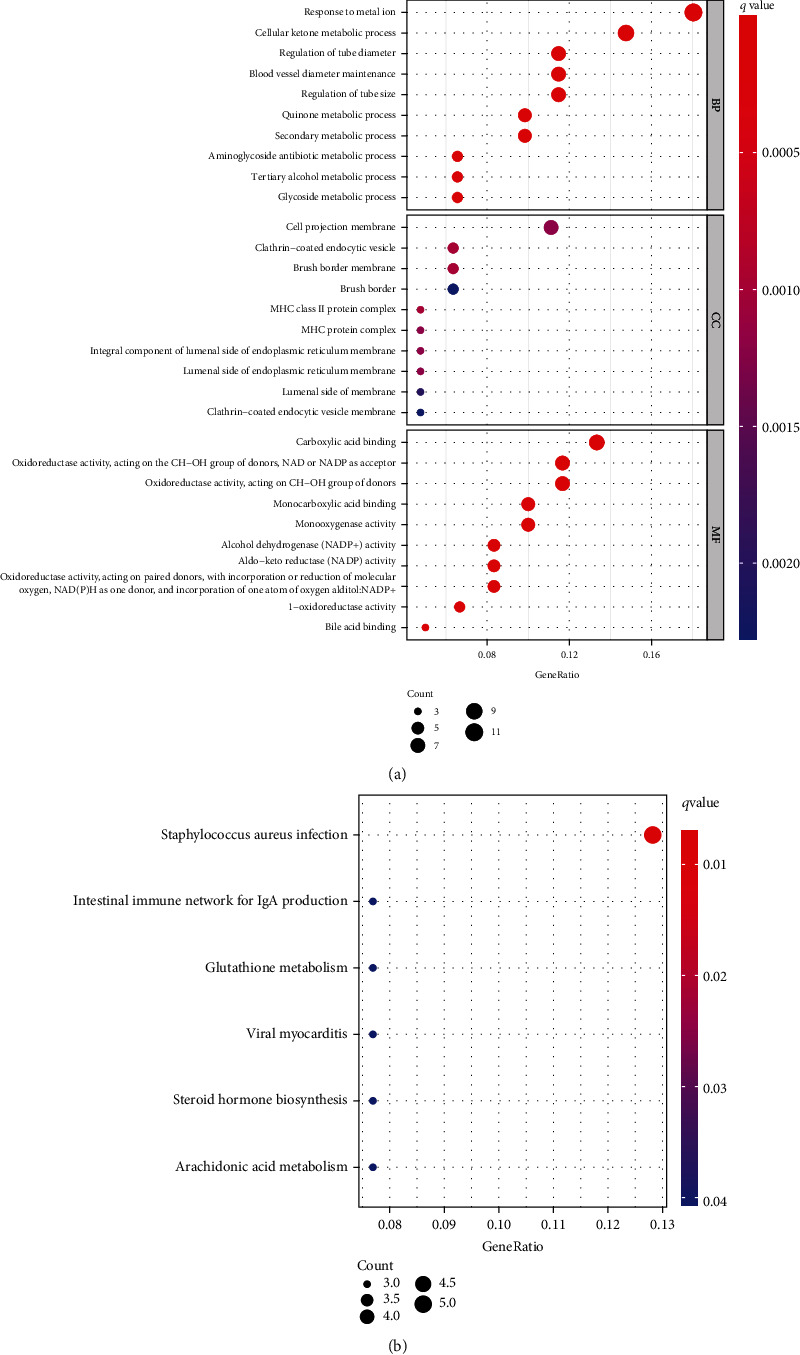
(a) GO analyses and (b) biological pathway enrichment analyses of the dysregulated genes.

To explore the function of SCL7A11 in LUAD, our team selected DEGs between LUAD specimens with high SCL7A11 expression and LUAD specimens with low SCL7A11 expression. The GO analyses revealed that the GO annotations of differentially expressed genes were separated into 3 parts: biological process (BP), cell composition (CC), and molecular function (MF). Terms were in an ascending order as per the FDR results. Posterior to the selection process, we discovered that (FDR<0.05) along with DEGs were concentrated in BP, like response to metal ion, cellular ketone metabolic process, cell projection membrane, clathrin-coated endocytic vesicle, carboxylic acid binding, and monocarboxylic acid binding (Figure 10(a)). The KEGG analyses revealed that DEGs were predominantly enriched in Staphylococcus aureus infection, intestine immunity network for IgA generation, glutathione metabolism, viral myocarditis, steroid hormone biosynthesis, and arachidonic acid metabolism (Figure 10(b)).

## 4. Discussion

Based on the Chinese statistics as to LUAD development, it is evident that LUAD prognosis factors preventing and curing LUAD require further exploration [20]. Investigating novel molecule-level prognosis and prediction biomarkers is a hotspot in medical researches [21, 22]. Recently, more and more researches have reported the underlying effects of ferroptosis-related genes utilized as new diagnosis and prognosis markers for LUAD [23–25]. Herein, our team identified 23 dysregulated ferroptosis-associated genes in LUAD. Among them, SLC7A11, GLS2, ATP5MC3, and FANCD2 were associated with five-year survivals of LUAD patients, suggesting their potential use as novel biomarkers.

Previously, several studies have reported the function of the above four genes in several types of tumors, including LUAD. For instance, nuclear and cytoplasmic localization of FANCD2 was identified in ovarian carcinoma from both healthy and ovarian carcinoma sufferers [26]. Sufferers with cytoplasm localization of FANCD2 had remarkably better median survival duration (50 months), in contrast to sufferers in the absence of cytoplasm localization of FANCD2. In addition, cytoplasm FANCD2 was discovered to be capable of binding protein participating in the native immune system, cell reaction to thermal stress, amyloid fiber formation, and estrogen-mediated signal transduction [27]. High ATP5MC3 expression predicted a poor prognosis in prostate cancer, colon cancer, and endometrial cancer [28–30]. TGF-*β*1-mediated inhibition of SLC7A11 drives vulnerability to GPX4 suppression in hepatic cell cancer [31]. In this study, we focused on SLC7A11 and found that high SLC7A11 expression predicted an advanced clinical pTNM_stage in LUAD patients. In clinical practice, pTNM_stage has been considered an important factor for the prediction of clinical outcome of LUAD patients, and its degree was strongly associated with distant metastasis [32, 33]. Thus, our findings suggested SLC7A11 may play a functional role in tumor metastasis. In addition, a survival study confirmed that high SLC7A11 expression predicted a poor prognosis in LUAD patients, which may be due to its potential function on tumor stage and metastasis.

With the development of cancers, our immunosystems are stimulated to inhibit tumorous progression [34]. It is unfortunate that oncocytes leverage a variety of methods to postpone or even prevent our immunosystems from repressing cancers, a phenomenon called immunoescape [35, 36]. The onset of immunoescape normally induces the development of malignancies, metastases, unsatisfactory prognoses, and immune therapy failures [37, 38]. Immune checkpoints, like programmed cell death protein-1 (PD-1), PD ligand-1 (PD-L1), and cytotoxic T-lymphocyte-associated protein 4 (CTLA4), account for immunoescape [39, 40]. Herein, our team discovered that eight immune checkpoints exhibited a dysregulated level in LUAD. Moreover, we found SLC7A11 expression was associated with several Immune checkpoints, indicating NLRP3 promoted immune escape in LUAD. Moreover, our team discovered that the expression of SLC7A11 was negatively associated with the expression of B cells and macrophage while being positively associated with that of T cell CD4+. Those associations imply an underlying causal link in which SLC7A11 modulates T cell functions in LUAD. Collectively, those discoveries reveal that the SLC7A11 is vital for recruiting and regulating immune infiltration cells in LUAD.

Nonetheless, there are certain deficiencies herein. Firstly, the present paper was a research finished retrospectively, although our team strove to involve as many data sets as possible to more strictly validate such hallmark. Further prospective researches were required to evidence the prognosis value of SLC7A11. Secondly, this report was restricted by the lack of experiment proofs. More researches exploring lineage cells and encompassing animal assays are warranted to substantiate the discoveries herein and unravel the molecule-level causal links.

## 5. Conclusion

In summary, our study suggested that four ferroptosis-related genes could be used as a prognostic indicator for LUAD with which to predict patient risk. In addition, SLC7A11 was related to immune therapy-associated biological markers, revealing its application significance in forecasting the potency of immune therapy.

## Data Availability

The datasets utilized and/or evaluated during this study are accessible upon valid request from the corresponding authors.
